# Effectiveness of Telemedicine on Wound-Related and Patient-Reported Outcomes in Patients With Chronic Wounds: Systematic Review and Meta-Analysis

**DOI:** 10.2196/58553

**Published:** 2025-06-10

**Authors:** Xiaoyan Zhang, Zhanghui Guo, Yu Duan, Chao Sun, Jiayin Luo

**Affiliations:** 1 Department of Vascular Surgery, Beijing Hospital, National Center of Gerontology, Institute of Geriatric Medicine, Chinese Academy of Medical Sciences, 1 Dahua Road, Dongdan, Dongcheng District, Beijing, China, 86 13718677576; 2 School of Nursing, Beijing University of Chinese Medicine, Beijing, China; 3 Department of Nursing, Beijing Hospital, National Center of Gerontology; Institute of Geriatric Medicine, Chinese Academy of Medical Sciences, Beijing, China

**Keywords:** telemedicine, chronic wound, systematic review, wound healing, meta-analysis, patient-reported outcome, randomized controlled trial

## Abstract

**Background:**

Telemedicine may provide new vitality and opportunities to the field of wound care and has been advocated as being a potential and feasible strategy for chronic wound management.

**Objective:**

This systematic review and meta-analysis aimed to assess the effectiveness of telemedicine on wound-related outcomes and patient-reported outcomes in patients with chronic wounds.

**Methods:**

A comprehensive search of 9 databases, including PubMed, Embase, PsycINFO, the Cochrane Library, CINAHL, Web of Science, the China National Knowledge Infrastructure database, the Wanfang database, and the VIP database, was performed to identify eligible randomized controlled trials that investigated the effectiveness of telemedicine for patients with chronic wounds. The primary outcome was wound healing, including healing score, healing time, and healing rate. The quality of the included studies was examined via the Cochrane risk-of-bias tool. Data synthesis was conducted via Review Manager (version 5.4; the Cochrane Collaboration). Due to anticipated heterogeneity, a random-effects meta-analysis was used. Effect estimates are presented as risk ratio (RR) or standard mean differences (SMDs) with 95% CI. The quality of the evidence was assessed via the Grading of Recommendations, Assessment, Development, and Evaluation approach.

**Results:**

A total of 22 randomized controlled trials involving 2397 participants met the inclusion criteria. This review demonstrated that telemedicine significantly improved the healing score (SMD −1.46, 95% CI −2.27 to −0.66; *P*<.001*; I*^2^=95%; *P*<.001), healing time (SMD −0.47, 95% CI −0.92 to 0.02; *P*=.04*; I*^2^=85%; *P*<.001), amputation rate (RR 0.52, 95% CI 0.31-0.88; *P*=.02; *I*^2^=23%; *P*=.28), pain (SMD−0.62, 95% CI −0.90 to −0.34; *P*<.001; *I*^2^=0%; *P*=.32), and quality of life (SMD 1.90, 95% CI 0.32-3.48; *P*=.02; *I*^2^=98%; *P*<.001). Although the meta-analysis results indicated that telemedicine enhanced the healing rate (RR 1.16, 95% CI 1.02-1.33; *P*=.03; *I*^2^=50%; *P*=.03), potential publication bias was detected (Egger test, bias=1.801; SE 0.367; *P*<.001). Upon imputing the missing studies using the trim-and-fill method, the recalculated pooled RR was adjusted, resulting in a new estimate of RR 1.06 (95% CI 0.98-1.15; *P*=.16). In addition, no significant differences were found in mortality, depression, anxiety, or patient satisfaction.

**Conclusions:**

There is some evidence that telemedicine contributes to improvements in the healing score, healing time, amputation rate, pain, and quality of life of patients with chronic wounds. Nevertheless, further high-quality studies are essential to examine the impact of telemedicine on healing rate and patient-reported outcomes in patients with chronic wounds.

## Introduction

### Background

A chronic wound is defined as a wound that fails to achieve anatomical and functional integrity through a normal and orderly repair process within 4 weeks [1,2]. Chronic wounds pose a significant global challenge and include conditions such as venous ulcers, arterial ulcers, diabetic foot ulcers, and pressure injuries. The global incidence of chronic wounds is approximately 2.21 per 1000 people [3]. According to previous studies, the treatment of chronic wounds accounts for between 3% and 5.5% of all medical expenses in high-income countries [4]. The cost of hospitalization for diabetes-related amputations ranges from US $12,851 to US $16,267 per admitted patient [5]. Additionally, the cost for leg ulcers ranges from US $44,900 to US $23,700 per patient for primary and outpatient care [5]. Moreover, patients with chronic wounds frequently experience discomfort, infection, anxiety, despair, and even the possibility of amputation. These factors profoundly impact the physical and mental well-being of patients as well as their quality of life [6]. As the aging population grows and the prevalence of chronic diseases (such as diabetes) continues to increase, the incidence and economic burden associated with chronic wounds are rapidly escalating [7,8].

Patients with chronic wounds typically experience complex conditions, thus necessitating long hospital stays averaging 13 days [9]. Due to constraints such as bed turnover rates and hospitalization costs, these patients often do not fully recover in hospital settings and must continue receiving care at home. This transition poses challenges, especially when primary care nurses and family caregivers lack adequate knowledge and resources for effective wound management. Over half of nurses have insufficient knowledge of treatment methods and wound care evaluation, and only 36.74% (n=313) can easily access essential information [10]. Family members, who spend approximately 10 hours daily on caregiving, are overwhelmed by the demands of home-based care [11]. This gap in continuous, effective management has led to frequent hospital readmissions, thus resulting in worsened patient despair and hopelessness. Therefore, addressing these issues via technological innovations in chronic wound management is critical.

The adoption of telemedicine has markedly increased since the COVID-19 pandemic, as evidenced by significant policy efforts being enacted throughout the world. In 2022, the World Health Organization released its Consolidated Telemedicine Implementation Guide [12], whereas the Registered Nurses Association of Ontario introduced the Clinical Practice in a Digital Health Environment guideline [13]. Similarly, China has advanced its digital health care through key policies such as the “14th Five-Year Plan” for Digital Economy Development (Guofa [2021] No 29) [14] and the “National Nursing Development Plan (2021‐2025)” (Guofa [2022] No 15) [15]. As a result, telemedicine is now a vital tool for both patients and a wide range of health care professionals. Telemedicine, which enables the distribution of health care services and information via electronic and telecommunications technologies, supports long-distance patient care, education, and monitoring [16,17]. Its applications are diverse and encompass diabetes management [18], exercise regimens for coronary heart disease [19], and stroke care [20]; moreover, it holds significant potential for managing chronic wounds. By facilitating remote diagnosis and treatment, telemedicine offers continuous care, particularly to patients in remote locations, thereby optimizing medical resource use [21]. It also allows health care providers to deliver targeted chronic wound care training and supervision via remote platforms, thus improving patient self-management and ensuring precise wound care [22,23].

### Objective

Given these advantages, telemedicine may provide new vitality and opportunities for the management of chronic wounds. Researchers have begun to verify its effectiveness and safety in the remote care of patients with chronic wounds. However, the sample size of a single study is small, and there are differences in the types of chronic wounds, forms of intervention, intervention time, number of types of telemedicine, and whether telemedicine is associated with a face-to-face component. The results of a systematic review [24] revealed that there was no significant difference in the efficacy or safety of telemedicine compared with traditional treatment methods; however, this study still had several limitations. First, this review searched only 3 databases and included only a few randomized controlled trials (RCTs), although more RCTs have been published in recent years [25-32]. Furthermore, this review reported only wound-related outcomes and did not summarize evidence related to patient-reported outcomes (PROs), such as pain, depression, anxiety, and quality of life. However, PROs play a crucial role in treating chronic wounds [33]. These outcomes can help researchers to understand patients’ attitudes and needs as well as comprehensively evaluate the role of interventions in improving patients’ overall health status [34]. Therefore, we aimed to conduct a systematic review and meta-analysis of RCTs to assess the effectiveness of telemedicine on wound-related outcomes and PROs in patients with chronic wounds.

## Methods

This study followed the PRISMA (Preferred Reporting Items for Systematic Review and Meta-Analyses) statement and checklist [35] (Checklist 1). The protocol of this review was registered in PROSPERO (CRD42023462475).

### Data Sources and Search Strategy

A 3-step search strategy was used. The first search was conducted in PubMed and the China National Knowledge Infrastructure database. The used search terms were located by analyzing keywords related to chronic wounds, telemedicine, and study design within titles and abstracts, as well as the index terms that were used to describe the papers. The second search was performed by using all of the identified search terms across 9 databases from their inception to the present time. These databases included PubMed, Embase, PsycINFO, the Cochrane Library, CINAHL, Web of Science, the China National Knowledge Infrastructure database, the Wanfang database, and the VIP database. The search strategy for each database was discussed by all of the reviewers with literature search expertise (see Multimedia Appendix 1 for the full search strategy). The third search was performed by reviewing the reference lists of the identified papers for additional relevant studies. Publications in English or Chinese were included, with no restrictions on the year of publication.

### Study Selection

The rationale for the selection of inclusion or exclusion criteria was based on the population, intervention, control, outcome, study design framework [36]: (1) population: individuals of all ages with chronic wounds, (2) intervention: telemedicine as the main intervention, (3) control: any control condition (eg, usual care, treatment as usual, or nontelemedicine interventions), (4) outcome: reported wound-related outcomes (eg, wound healing, amputation rate, mortality, and economic evaluation) or PROs (eg, pain, quality of life, depression, anxiety, and patient treatment and care satisfaction). Wound healing was the primary outcome, others were the secondary outcomes. While our registered protocol initially included sleep as a PRO, this outcome was subsequently excluded, as it was not central to our primary research questions, and none of the included studies reported sleep-related outcomes; and (5) study design: RCTs. The exclusion criteria were as follows: (1) studies identified as protocols and (2) studies not published in English or Chinese. Study selection was conducted in 2 distinct stages. In the first stage, 2 reviewers (ZG and YD) independently screened all titles and abstracts against the predefined inclusion and exclusion criteria. In the second stage, full-text papers that potentially met the inclusion criteria were retrieved and independently evaluated by 1 reviewer (ZG) who assessed all papers, while the second reviewer (YD) independently assessed a randomly selected 30% (n=22) subset of full texts. If disagreements were identified in 2 or more papers within this 30% (n=22) subset, the second reviewer would then assess the remaining 70% (n=22) of papers to ensure methodological rigor. Throughout both stages, all disagreements between the 2 reviewers were documented and resolved through discussion. When consensus could not be reached, a third reviewer (JL) was consulted to make the final determination regarding study inclusion.

### Data Extraction

The data from the included studies were extracted by ZG and then cross-checked by YD. Any discrepancies were resolved by a third reviewer (CS). For included RCTs, we extracted the following data as recommended in the Cochrane Handbook for Systematic Reviews of Interventions [37]: (1) general information: author, country, publication language, publication year, and journal citation; (2) participants: inclusion and exclusion criteria, sample size, baseline characteristics, and setting; (3) interventions: details of telemedicine according to the TIDieR (Template for the Intervention Description and Replication)-telehealth checklist [38]; (4) risk of bias in trials; (5) follow-up: length of follow-up, the reason for and number of dropouts and withdrawals, and method of analysis; and (6) outcome measures: mean and SD for continuous outcomes and the number of events for dichotomous outcomes. In instances where the data were missing, unclear, or incomplete, attempts were made to contact the authors via email for further clarification. In cases where the SD was not reported with means and the necessary information was not obtained from the trial authors, it was imputed based on the information provided, such as the SE, 95% CI, or *P* values, following the guidelines in the *Cochrane Handbook*. Alternatively, if the SD for the missing outcome was not available, it was assumed to be the average of the SDs from trials where this information was reported.

### Risk of Bias

The methodological quality of the included studies was independently assessed by 2 reviewers (XZ and JL) via version 2 of the Cochrane tool to assess the risk of bias in randomized trials [39]. It included 5 domains: “randomization process,” “deviations from intended interventions,” “missing outcome data,” “measurement of the outcome,” and “selection of the reported result.” Any disagreements were resolved by a third reviewer (JL).

### Statistical Analysis

Pooled analyses were performed using Review Manager (version 5.4; The Cochrane Collaboration). Given the anticipated high levels of heterogeneity due to the diverse forms of telemedicine, the meta-analyses were based on the random‐effects model to obtain the most conservative result. Where available, analyses were based on intention-to-treat data from the individual trials. Furthermore, when possible, end-of-treatment scores (rather than change-from-baseline scores) were extracted for the continuous outcomes. Dichotomous data are presented as the risk ratio (RR) and 95% CI, whereas continuous variables are expressed as the mean difference and 95% CI. Standardized mean differences (SMDs) were calculated for continuous outcomes that were measured or reported differently. Narrative synthesis was performed when the quantitative synthesis was judged to be unsuitable.

Heterogeneity was assessed via the Cochran *Q* test and *I*^2^ statistics. If substantial heterogeneity was found (*I*^2^ of 50% or more and a *P* value of *Q* test<.10), the possible causes by undertaking a prespecified subgroup analysis were reported and investigated. Planned subgroup analyses (where applicable) were performed to compare effect estimates between studies based on the following criteria: (1) the average age of participants (ie, 18‐60 or ≥60 years), (2) study duration (ie,≤6, 6‐12, or>12 months), (3) number of types of telemedicine (ie, 1 or ≥2), (4) types of chronic wounds (ie, mixed chronic wounds, diabetic foot ulcers, stress injuries, or static ulcers), (5) whether telemedicine was used in conjunction with a face-to-face component (ie, yes or no), and (6) whether telemedicine included a communication component (ie, yes or no). Sensitivity analyses were performed for primary outcomes to determine whether the conclusions were robust to arbitrary decisions made regarding eligibility and analysis. When substantial heterogeneity existed, sensitivity analysis was also conducted to further investigate the potential sources of heterogeneity. Potential publication bias was evaluated by using funnel plots and the Egger test using R statistical software (version 4.4.2; R Foundation for Statistical Computing) when sufficient studies were available. If publication bias was detected, a trim-and-fill method was used to adjust for publication bias. Two reviewers (XZ and JYL) assessed the certainty of evidence for hospitalization using the Grading of Recommendations, Assessment, Development, and Evaluation approach. Any disagreements were resolved by the third reviewer (JL).

## Results

### Overview

**Figure 1.**
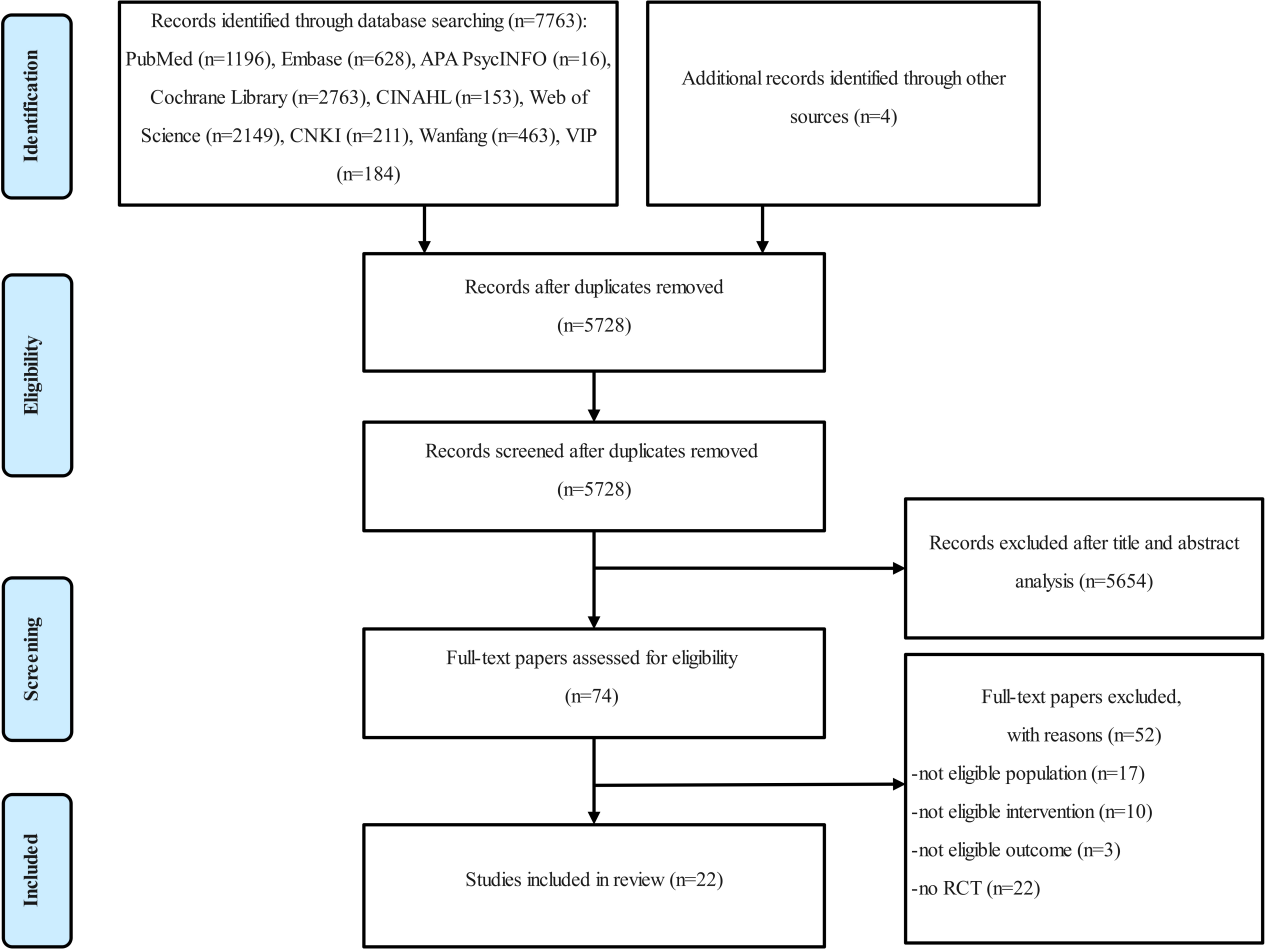
Flow diagram of the study screening process. CNKI: China National Knowledge Infrastructure; RCT: randomized controlled trial.

A total of 7767 records were retrieved from the initial database search and reference lists of key papers. Ultimately, 22 RCTs [25-32,40-53] involving 2397 patients met the inclusion criteria. Figure 1 displays the PRISMA flow diagram.

### Study Characteristics

The basic characteristics of the included studies are presented in Multimedia Appendix 2 [25-32,40-53]. These studies were published between 2004 and 2023, with 12 studies conducted in China [25-28,31,32,40,41,44,46,47,50], 2 in the United States [51,52], 2 in Australia [43,53], 2 in Norway [29,42], 1 in Denmark [45], 1 in Canada [48], 1 in France [30], and 1 in the United Kingdom [49]. Among the studies, 11 [25,28,30,31,40,41,49-53] focused on mixed chronic wounds, 5 [26,27,29,42,45] focused on diabetic foot ulcers, 5 [32,43,44,46,48] focused on stress injuries, and 1 [47] focused on static ulcers. Various telemedicine methods were used across the studies, including mobile apps (eg, wound management systems, WeChat, and Tencent QQ), remote robots, web-based interventions, emails, phone calls, and multimedia messaging services (see Multimedia Appendix 2 for details). A total of 10 studies [25,28,30,31,40,43,49,50,52,53] included 1 telemedicine component, and the remaining 12 studies [26,27,29,32,41,42,44-48,51] used 2 or more telemedicine components. In addition, 19 studies [25-32,40-48,51,52] integrated telemedicine with a communication component, and 13 studies [25-28,32,40-42,44-46,48,52] involved telemedicine in conjunction with a face-to-face component. The intervention duration varied among the included studies and ranged from 2 weeks to 1 year. In total, 15 studies [25,27,28,30-32,40,41,43,44,46,47,49,50,52] had a short duration (≤6 months), 6 studies [26,29,42,45,48,53] had a medium duration (>6 to ≤12 months), and 1 study [51] had a long duration (>12 months). A total of 15 interventions [25,29,30,32,40,42-45,48-53] were delivered individually, 6 [27,28,31,41,46,47] were delivered in a mixed manner, and 1 [26] was delivered in a group. Most of the telemedicine procedures involved tailoring interventions via individualized assessments, individualized guidance, individualized question answering, or individualized strategies. The comparison conditions included usual care, standard care, traditional care, and nontelemedicine components. The number of participants in the included studies ranged from 26 to 374, and their mean age ranged from 35 to 83 years. A total of 13 studies [28-32,40,42,44-46,48,49,53] included a slightly older participant population, with an average age of ≥60 years observed in both groups. All of the reported outcomes, including wound-related outcomes and PROs, were measured after treatment.

### Risk of Bias

A total of 21 studies [25-32,40-49,51-53] were categorized as having “some concerns” in terms of the overall risk of bias, whereas 1 study [50] was deemed to have a “high risk.” All of the studies were designated as “randomized,” with 13 studies [25,26,28,29,31,40-44,47-49] using a computer-generated random number generator or a random number table. In contrast, other studies [27,30,32,45,46,51,52] only mentioned random allocation and did not clarify the allocation method. Given the nature of the telemedicine, participants’ awareness of their assigned interventions during the trial was plausible, and a lack of information about whether researchers blinded the methods resulted in a high judgment of “some concerns.” All of the studies [25-32,40-53] included the outcome data of the vast majority of the subjects, and the analysis results were robust. Thus, all of the studies were assessed as having a low risk of bias. However, no studies provided sufficient information to determine whether the blinding of outcome assessments was achieved, thus leading to the assumption that the assessors may have been aware of the interventions received by the study participants. Thus, all of the studies [25-32,40-53] had “some concerns” regarding the measurements of the outcomes. Six studies [29,30,42,43,45,48] registered study protocols, reported prespecified outcomes, and were assessed to be at low risk of reporting bias. A total of 16 studies [25-28,31,32,40,41,44,46,47,49-53] did not have a protocol available, thus resulting in “some concerns” about the risk of bias in the selection of the reported results (see Multimedia Appendix 3 [25-32,40-53] for more details).

### Effects of Telemedicine

#### Wound Healing

**Figure 2.**
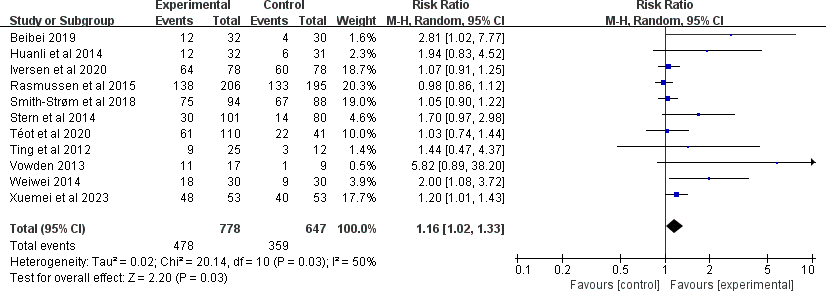
Forest plot of the effect of telemedicine on wound healing rate [25,29,30,41,42,45-50].

A total of 13 studies [25,29,30,41,42,45-51,53] involving 1631 participants reported the wound healing rate. Among these, 2 studies [51,53] were excluded from the quantitative synthesis because of the absence of an exact number of healed wounds or an uneven distribution of wound severity among groups. After pooling, telemedicine significantly increased the healing rate (RR 1.16, 95% CI 1.02-1.33; *P*=.03), with substantial heterogeneity observed between the studies (*I*^2^=50%; *P*=.03; Figure 2). The quality of the evidence was rated as being low because of imprecision and publication bias (see Multimedia Appendix 4 for more details). Subgroup analyses were further performed (see Multimedia Appendix 5 for more details). Short-term telemedicine [25,30,41,46,47,49,50] significantly increased the healing rate (≤6 months; RR 1.47, 95% CI 1.07-2.00; *P*=.02), whereas moderate-term [29,42,45] and long-term [48] telemedicine did not statistically increase the healing rate. Regarding the types of chronic wounds, combining telemedicine for pressure injuries [46,48] (RR 1.77, 95% CI 1.11-2.82; *P*=.02) or venous ulcers [47] (RR 2.00, 95% CI 1.08-3.72; *P*=.03) had a greater effect on the healing rate than diabetic foot ulcers [29,42,45] or mixed chronic wounds [25,30,41,49,50]. The combination of telemedicine in conjunction with a face-to-face component [25,41,42,45-48] had a greater effect on increasing the healing rate (RR 1.22, 95% CI 1.02-1.47; *P*=.03) than the absence of face-to-face components [29,30,49,50].

**Figure 3.**
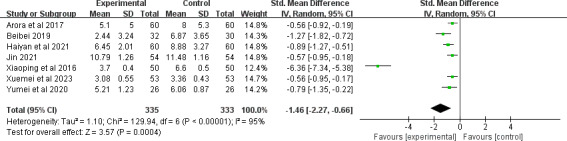
Forest plot of the effect of telemedicine on healing score [25,31,32,40,41,43,44].

A total of 8 studies [25,31,32,40,41,43,44,50] comprising 705 participants reported the healing score; 7 studies [25,31,32,40,41,43,44] used the pressure ulcer scale for healing, and 1 study [50] used a self-designed questionnaire. The study [50] that reported positive results was excluded from the quantitative synthesis due to the use of a different scoring approach. After pooling, telemedicine significantly improved the healing score (SMD −1.46, 95% CI −2.27 to −0.66; *P*<.001), with substantial heterogeneity observed between the studies (*I*^2^=95%; *P*<.001; Figure 3). The quality of the evidence was rated as being moderate because of inconsistency (see Multimedia Appendix 4 for more details). Subgroup analyses were further performed (see Multimedia Appendix 5 for more details). All of the subgroup analyses by average age, type of chronic wound, number of types of telemedicine, and whether the telemedicine was associated with a face-to-face component revealed statistically significant differences.

**Figure 4.**
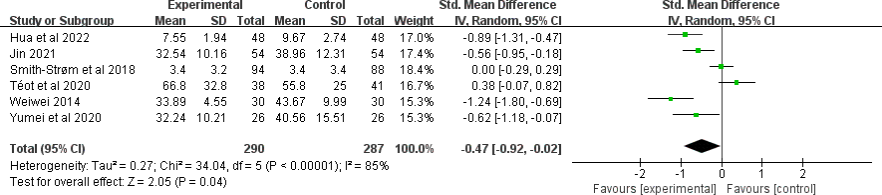
Forest plot of the effect of telemedicine on healing time [28,30,31,40,42,47].

A total of 8 studies [28,30,31,40,42,43,47,51] with 810 participants reported the healing time. Among these, 2 studies [43,51] were excluded from the quantitative synthesis because of the absence of an exact healing time or uneven distribution of the severity of the wounds among the groups. After pooling, the evidence suggested that telemedicine decreased the healing time (SMD −0.47, 95% CI −0.92 to −0.02; *P*=.04), although substantial heterogeneity existed among the studies (*I*^2^=85%; *P*<.001; Figure 4). The quality of the evidence was rated as being low because of inconsistency and imprecision (see Multimedia Appendix 4 for more details). Subgroup analyses were further performed (see Multimedia Appendix 5 for more details). Combining telemedicine for venous ulcers [47] (SMD −1.24, 95% CI −1.80 to −0.69; *P*<.001) had a greater effect size on the healing rate than those for diabetic foot ulcers [42] or mixed chronic wounds [28,30,31,40]. Combining telemedicine in conjunction with a face-to-face component [28,40,42,47] indicated a greater effect size for increasing healing time (SMD −0.66, 95% CI −1.25 to −0.08; *P*=.03) than those with no face-to-face components [30,31].

#### Amputation Rate

**Figure 5.**
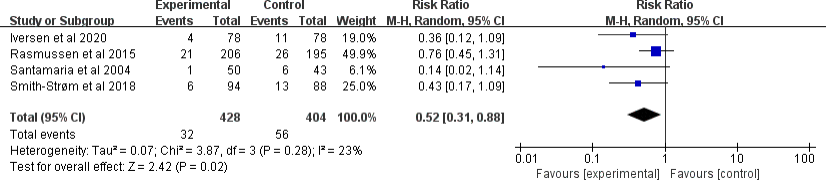
Forest plot of the effect of telemedicine on amputation rate [29,42,45,53].

A total of 4 studies [29,42,45,53] comprising 832 participants reported the amputation rate. After pooling, telemedicine significantly decreased the amputation rate (RR 0.52, 95% CI 0.31-0.88; *P*=.02) with small and nonsignificant heterogeneity between studies (*I*^2^=23%; *P*=.28; Figure 5). The quality of the evidence was rated as moderate due to the imprecision (see Multimedia Appendix 4 for more details). Subgroup analyses were further performed (see Multimedia Appendix 5 for more details). Combining studies [29,42,45] with more telemedicine types had greater effects on the amputation rate (RR 0.60, 95% CI 0.39-0.93; *P*=.02) than those with 1 telemedicine type [53]. Concerning the types of chronic wounds, combining telemedicine for diabetic foot ulcers [29,42,45] (RR 0.60, 95% CI 0.39-0.93; *P*=.02) had a greater effect size on amputation rate than mixed chronic wounds [53]. Furthermore, combining telemedicine with communication components [29,42,45] had a greater effect size on decreasing the amputation rate (RR 0.60, 95% CI 0.39-0.93; *P*=.02) than telemedicine without communication components [53].

#### Mortality

**Figure 6.**
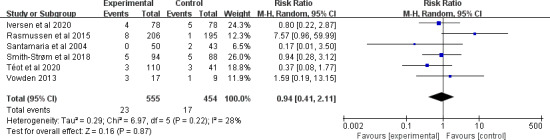
Forest plot of the effect of telemedicine on mortality [29,30,42,45,49,53].

A total of 6 studies [29,30,42,45,49,53] involving 1009 participants reported the outcome of mortality. After pooling, the evidence suggested that there was no statistically significant difference in the outcome of mortality (RR 0.94, 95% CI 0.41-2.11; *P*=.87), with small and nonsignificant heterogeneity observed between the studies (*I*^2^=28%; *P*=.22; Figure 6). The quality of the evidence was rated as being moderate due to imprecision (see Multimedia Appendix 4 for more details). Subgroup analyses were further performed (see Multimedia Appendix 5 for more details). Subgroup analysis by the study duration, number of types of telemedicine, type of chronic wounds, whether telemedicine was associated with a face-to-face component, and whether telemedicine included communication components revealed no statistically significant subgroup differences.

#### Economic Evaluation

A total of 6 studies [30,43,45,48,51,53] mentioned a cost analysis for the telemedicine intervention versus the control intervention. Santamaria et al [53] reported total cost savings of US $191,935 in the telemedicine group compared with the control group; however, the cost of ongoing management and support for amputees was not included in the analysis. Téot et al [30] demonstrated reduced costs associated with transport for wound management. Rasmussen et al [45,54] indicated that telemedicine follow-up was US $2300 less per patient than standard care, although this difference was not statistically significant (*P*=.42). Arora et al [43,55] reported that the mean total cost per participant in the intervention group was US $2460 compared with US $2391 for the control group. The per-participant cost of delivering the intervention was US $119. Another study [48] revealed that the economic evaluation demonstrated a mean reduction in direct care costs of US $650 per patient compared with the control group. Additionally, 1 study [51] revealed a higher mean total cost per patient due to greater and more severe ulcers in the telemedicine group.

#### Pain

**Figure 7.**
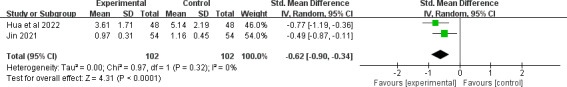
Forest plot of the effect of telemedicine on pain [28,31].

A total of 4 studies [28,31,48,50] involving 465 participants reported pain outcomes. Among these, 3 studies used the visual analog scale, whereas 1 study used a self-designed questionnaire. Two studies [48,50] were not included in the quantitative synthesis because of the absence of exact data on pain or different scoring approaches. Ting et al [50] reported that telemedicine significantly improved pain compared with the control group (*P*=.004). Stern et al [48] reported that the mean visual analog scale score for wound-specific pain was estimated to be 0.39 units higher; however, the difference was not statistically significant (*P*=.42). Furthermore, after pooling, a significant difference in the outcome of pain was detected between the 2 groups (SMD −0.62, 95% CI −0.90 to −0.34; *P*<.001; Figure 7). The quality of the evidence was rated as being moderate because of imprecision (see Multimedia Appendix 4 for more details).

#### Quality of Life

**Figure 8.**
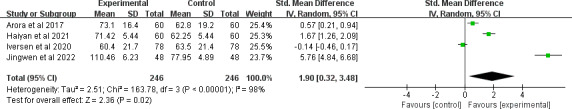
Forest plot of the effect of telemedicine on quality of life [27,29,32,43].

A total of 8 studies [26-29,32,40,41,43] comprising 784 participants reported quality of life outcomes. Among these, 4 studies [27,29,32,43] used the Short Form 36-item Health Survey, whereas the remaining 4 used the European Quality of Life-5 dimensions Questionnaire [29,43], the Diabetes-Specific Quality of Life Scale [27], and the World Health Organization Quality of Life-Brief Version [32]. Four studies [27,29,32,43] indicated that telemedicine significantly increased the overall quality of life (SMD 1.90, 95% CI 0.32-3.48; *P*=.02; Figure 8). The quality of the evidence was rated as being moderate because of inconsistency (see Multimedia Appendix 4 for more details). Additionally, the remaining 4 studies [26,28,40,41] reported Short Form 36-item Health Survey scores in each dimension. After pooling, telemedicine significantly increased physical functioning (3 RCTs, SMD 7.70, 95% CI 3.91-11.49; *P*<.001), role-physical (4 RCTs, SMD 2.46, 95% CI 0.46-4.46; *P*=.02), bodily pain (4 RCTs, SMD 3.87, 95% CI 1.34-6.40; *P*=.003), general health (4 RCTs, SMD 6.68, 95% CI 2.55-10.81; *P*=.002), vitality (4 RCTs, SMD 9.54, 95% CI 4.29-14.79; *P*=.001), social functioning (4 RCTs, SMD −0.80, 95% CI −2.11 to 0.51; *P*=.23), role-emotional (3 RCTs, SMD 4.61, 95% CI 1.19-8.04; *P*=.008), and mental health (4 RCTs, SMD 4.32, 95% CI 2.69-5.96; *P*<.001). However, no significant difference was detected in the outcome of the social functioning dimension of the 2 groups.

#### Depression

**Figure 9.**
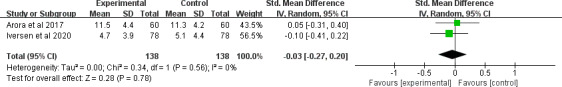
Forest plot of the effect of telemedicine on depression [29,43].

A total of 2 studies [29,43] involving 276 participants reported the outcome of depression, as measured via the hospital anxiety and depression scale. After pooling, no significant difference was detected in the outcome of depression between the 2 groups (SMD −0.03, 95% CI −0.27 to 0.20; *P*=.78; Figure 9). The quality of the evidence was rated as being moderate due to imprecision (see Multimedia Appendix 4 for more details).

#### Anxiety

**Figure 10.**
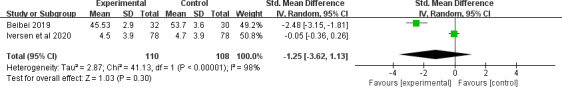
Forest plot of the effect of telemedicine on anxiety [29,41].

A total of 2 studies [29,41] with 218 participants reported outcomes of anxiety. Among these, 1 study [41] used the self-rating anxiety scale, and 1 study [29] used the hospital anxiety and depression scale. After pooling, no significant difference in the outcome of anxiety was detected between the 2 groups (SMD −1.25, 95% CI −3.62 to 1.13; *P*=.30; Figure 10). The quality of the evidence was rated as being low because of inconsistency and imprecision (see Multimedia Appendix 4 for more details).

#### Patient Satisfaction

**Figure 11.**
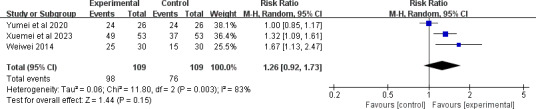
Forest plot of the effect of telemedicine on patient satisfaction rate [25,40,47].

A total of 3 studies [25,40,47] involving 174 participants reported the outcome of the patient satisfaction rate. After pooling, no significant difference was detected in the patient satisfaction rate between the 2 groups (RR 1.26, 95% CI 0.92-1.73; *P*=.15; Figure 11). The quality of the evidence was rated as being low due to inconsistency and imprecision (see Multimedia Appendix 4 for more details).

**Figure 12.**
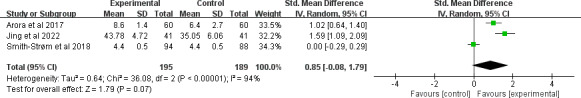
Forest plot of the effect of telemedicine on patient satisfaction score [26,42,43].

A total of 3 studies [26,42,43] comprising 174 participants reported the outcome of patient satisfaction scores. After pooling, no significant difference was detected in patient satisfaction scores between the 2 groups (SMD 0.85, 95% CI −0.08 to 1.79; *P*=.07; Figure 12). The quality of the evidence was rated as being low due to inconsistency and imprecision (see Multimedia Appendix 4 for more details). Additionally, 1 study [52] reported that telemedicine serves as a valuable aid in increasing the rate of satisfaction, with care decisions ultimately being made during direct consultations (*P*<.01).

### Sensitivity Analysis

A total of 6 sensitivity analyses were conducted to explore possible sources of heterogeneity and assess the robustness of the results. The removal of 1 study [49] with a small sample explained part of the substantial heterogeneity in the healing rate (*I*^2^=49%; *P*=.04) but did not alter the significance or direction of the effect (SMD 1.14, 95% CI 1.01-1.29; *P*=.04). The removal of 1 study [44] that used a different measurement explained the most substantial heterogeneity in healing sores (*I*^2^=25%; *P*=.25), but the significance or direction of the effect remained unchanged (SMD −0.73, 95% CI −0.93 to −0.53; *P*<.001). The removal of 1 study [30] that delivered an intervention through a dedicated cloud-based software program also explained part of the substantial heterogeneity in healing time (*I*^2^=82%; *P*=.001). However, the significance or direction of the effect did not change (SMD −0.64, 95% CI −1.08 to −0.19; *P*=.005). The removal of 1 study [27] that delivered the intervention through an app explained a small amount of substantial heterogeneity in the quality of life (*I*^2^=96%; *P*<.001), and the significance and direction of the effect changed (SMD 0.69, 95% CI −0.32 to 1.71; *P*=.18). The removal of 1 study [40] with patients with an older average age explained the most substantial heterogeneity in the patient satisfaction rate (*I*^2^=14%; *P*=.28), resulting in a change in the significance and direction of the effect (SMD 1.40, 95% CI 1.15-1.71; *P*=.001). Similarly, the removal of 1 study [42] with patients with an older average age also explained part of the substantial heterogeneity in patient satisfaction scores (*I*^2^=69%; *P*=.07), leading to a change in the significance and direction of the effect (SMD 1.28, 95% CI 0.72-1.84; *P*<.001).

### Publication Bias

The funnel plots (n>10) for the outcomes of the healing rate were asymmetrical (Egger test, bias=1.801; SE 0.367; *P*<.001), thus indicating the presence of a potential bias (see Multimedia Appendix 6 funnel plot for more details). The trim-and-fill test revealed 5 missing studies (see Multimedia Appendix 7 funnel plot for more details). The addition of the missing studies to the left part of the funnel plot changed the effect size (RR 1.06, 95% CI 0.98-1.15; *P*=.16). Moreover, no significant publication bias was found for the outcome of healing score, healing time, and mortality (n>5; Egger test, all *P*>.05).

## Discussion

### Main Findings

This review aimed to evaluate the effectiveness of telemedicine in chronic wound management. A total of 22 RCTs involving 2397 participants met the inclusion criteria. Overall, the use of telemedicine significantly improved the healing time, healing score, amputation rate, pain, and quality of life of patients with chronic wounds. Subgroup analyses revealed significant effects of short-term telemedicine, telemedicine for pressure injuries or venous ulcers, and telemedicine in conjunction with a face-to-face component on improving the healing rate. Subgroup analyses revealed a significant decrease in healing time with telemedicine for venous ulcers or telemedicine in conjunction with a face-to-face component. Subgroup analyses also revealed significant effects of telemedicine with ≥2 telemedicine types, telemedicine for diabetic foot ulcers, or telemedicine in combination with communication components in improving the amputation rate. In conclusion, telemedicine appears to be an effective method for chronic wound management.

### Interpretation of Results

An important finding of this review is that telemedicine can significantly improve chronic wound healing, particularly in terms of reducing healing time (SMD −0.47, 95% CI −0.92 to −0.02; *P*=.04) and improving the healing score (SMD −1.46, 95% CI −2.27 to −0.66; *P*=.001). This effect is likely attributable to telemedicine’s ability to provide real-time, efficient remote monitoring, diagnosis, and treatment, which ensures timely and professional medical intervention, thereby optimizing wound management and accelerating the healing process. However, these findings contrast with those of a previous study [56], in which telemedicine was shown to have no significant effect on healing time. One explanation for this result is that the previous meta-analysis included only 4 RCTs with 254 participants, whereas our result was based on 6 RCTs with 577 participants. A greater sample size would increase confidence in our findings. Furthermore, our study demonstrated that the healing score in the telemedicine group was significantly higher than in the control group. The healing score encompasses critical factors such as wound area, fluid volume, and tissue type, which are integral to the improvement of wound healing time.

Moreover, the evidence in this review suggested that the healing rate of chronic wounds in the telemedicine group was 1.16 times greater than that in the control group (RR 1.16, 95% CI 1.01-1.33; *P*=.03). This result contrasts with the outcome of a prior meta-analysis conducted by Chen et al [24] (RR 1.21, 95%CI 0.96-1.53; *P*=.11). However, potential publication bias was observed in our study (Egger test, bias=1.801; SE 0.367; *P*<.001). After imputing missing studies by using the trim-and-fill method, the recalculated pooled RR was changed (RR 1.06, 95% CI 0.98-1.15; *P*=.16). Therefore, this result needs to be interpreted with caution and should be verified by more studies in the future. Additionally, this review revealed that the impact of telemedicine on healing rates may differ according to the intervention duration and the type of chronic wound. Patients with chronic wounds may benefit from the implementation of telemedicine in the short term, thus indicating that the effect of telemedicine on wound healing rates is more pronounced in the early stages. Concerning types of chronic wounds, combining telemedicine for pressure injuries or venous ulcers had a greater effect on the healing rate than telemedicine for diabetic foot ulcers or mixed chronic wounds. However, due to the relatively small number of studies included in these subgroups, these findings also should be interpreted with caution.

This review demonstrated that telemedicine significantly reduced the amputation rate compared to the control interventions, thus corroborating findings from another meta-analysis [24]. Telemedicine real-time remote monitoring and treatment technologies enable more accurate and timely interventions for chronic wounds, thus reducing the risk of amputation due to treatment delays or errors. Furthermore, the subgroup analysis suggested that ≥2 types of telemedicine had a greater effect on the amputation rate than 1 type of telemedicine. This enhanced efficacy likely results from the comprehensive and precise management strategies enabled by the integration of multiple technologies. Subgroup analysis indicated that combining telemedicine for diabetic foot ulcers had a greater effect on the amputation rate than telemedicine for mixed chronic wounds. This finding can be explained by the fact that patients with diabetes have a 15- to 20-fold greater risk of amputation than patients without diabetes [57-59]. Moreover, the subgroup analysis revealed that telemedicine including communication components had a greater effect on decreasing the amputation rate than telemedicine with no communication components. This is supported by a Cochrane review [60], which highlighted the benefits of interactive telemedicine in improving health care outcomes and professional practices.

Notably, this review indicates that, compared with telemedicine alone, combining telemedicine with face-to-face components significantly enhances healing rates and reduces healing time. Although telemedicine provides convenient and timely assessments, adding face-to-face interactions allows wound therapists to perform more comprehensive and detailed assessments. Direct observation and tactile examinations during these interactions offer additional insights into the wound’s condition, the health of the surrounding tissues, and the patient’s overall health, thus enabling more accurate treatment planning. Moreover, face-to-face interactions facilitate direct communication between patients and therapists, thus enhancing patients’ understanding of and trust in the treatment plan, which can correspondingly improve compliance. These findings suggest that, in practical terms, a combination of remote and face-to-face management can be effective for chronic wound management.

Concerning the PROs in this review, telemedicine significantly decreased pain compared with the control group. Telemedicine overcomes geographical limitations and facilitates patients’ access to continuous care from a professional medical team and personalized pain management solutions, thus effectively alleviating pain symptoms. Additionally, this review demonstrated evidence that telemedicine significantly improved the quality of life compared with that of the control group. Numerous studies [61,62] have illustrated the potential of telemedicine to increase patients’ quality of life. Regrettably, the robustness of the results of this study was compromised in the sensitivity analysis. The removal of 1 study [27] that delivered interventions through an app explained a small amount of the substantial heterogeneity in the quality of life, and the significance and direction of the effect changed. Similarly, the findings regarding whether telemedicine can enhance patient satisfaction are also not robust. Hence, future research is needed to investigate the impacts of telemedicine on the quality of life and satisfaction of patients with chronic wounds.

### Strengths and Limitations

This review has notable strengths. First, our conclusions were grounded in the best available evidence because we included only RCTs. Second, the study’s robustness was enhanced by subgroup and sensitivity analyses. Third, we included both wound-related outcomes and PROs to provide a comprehensive assessment of patients with chronic wounds. However, several limitations warrant acknowledgment. First, the methodological quality of the included studies varied, with some exhibiting relatively low quality, which may have influenced the meta-analysis results. Second, publication bias may be present, although we refrained from using a funnel plot for most outcomes due to the insufficient number of studies. Nevertheless, we mitigated potential publication bias by using a comprehensive search strategy. Third, only a randomly selected 30% (n=22) of full-text papers underwent dual independent assessment. While this approach could potentially introduce subjective interpretation risk, several factors mitigate this concern: we implemented a rigorous protocol with clear criteria; the dual review of the randomly selected 30% (n=22) sample showed complete agreement between reviewers, suggesting consistent application of inclusion or exclusion criteria. Fourth, language bias may have occurred due to limitations in the literature retrieval based on language. Fifth, we were unable to draw definitive conclusions regarding the potential of telemedicine to reduce costs, depression, and anxiety in patients with chronic wounds due to inconsistent results, the inability to pool data for meta-analysis, and the limited number of available studies. Despite these uncertainties, the inconclusive findings regarding these outcomes offer crucial insights that can guide future research in selecting appropriate outcome indicators. We encourage further investigations to comprehensively explore the effectiveness of telemedicine in enhancing PROs in individuals with chronic wounds.

### Conclusions

This review provides evidence supporting the significant enhancement of the healing time, healing score, amputation rate, pain, and quality of life of patients with chronic wounds through the application of telemedicine compared to the control group. Moreover, subgroup analyses revealed significant effects of short-term telemedicine, telemedicine for pressure injuries or venous ulcers, and telemedicine in conjunction with a face-to-face component on improving the healing rate. Subgroup analyses revealed a significant decrease in healing time with telemedicine for venous ulcers or telemedicine in conjunction with a face-to-face component. Subgroup analyses also revealed significant effects of telemedicine with ≥2 telemedicine types, telemedicine for diabetic foot ulcers, or telemedicine in combination with communication components in improving the amputation rate. Hence, telemedicine appears to be an effective method for chronic wound management, with future studies needed to explore their impact on healing rate and PROs in patients with chronic wounds.

## Supplementary material

10.2196/58553 Multimedia Appendix 1 Search strategy.

10.2196/58553 Multimedia Appendix 2 Characteristics of included studies.

10.2196/58553 Multimedia Appendix 3 Risk of bias of the included studies.

10.2196/58553 Multimedia Appendix 4 Grading of Recommendations, Assessment, Development, and Evaluation form of evidence certainty for telemedicine versus control group.

10.2196/58553 Multimedia Appendix 5 Subgroup analyses of effectiveness of telemedicine on wound-related and patient self-reported outcomes in patients with chronic wounds.

10.2196/58553 Multimedia Appendix 6 Funnel plot (effectiveness of telemedicine on healing rate).

10.2196/58553 Multimedia Appendix 7 Funnel plot after trim-and-fill method (effectiveness of telemedicine on healing rate).

10.2196/58553 Checklist 1 PRISMA (Preferred Reporting Items for Systematic Review and Meta-Analyses) checklist.

## Abbreviations

PRISMA: Preferred Reporting Items for Systematic Review and Meta-Analyses
PRO: patient-reported outcome
RCT: randomized controlled trial
RR: risk ratio
SMD: standard mean difference
TIDieR: Template for the Intervention Description and Replication
