# Supplementary material for: Effectiveness of Telemedicine on Wound-Related and Patient-Reported Outcomes in Patients With Chronic Wounds: Systematic Review and Meta-Analysis
Source: JMIR Mhealth Uhealth. 2025 Jun 10;13:e58553. doi: 10.2196/58553 (PMC12173094; doi:10.2196/58553)
Supplement: Multimedia Appendix 1 [file mhealth-v13-e58553-s001.doc]

Search results for each source

| **Database** | **No. refs found** | **Date searched** | **Searched by** |
| --- | --- | --- | --- |
| PubMed | 1196 | 11/9/2023 | XYZ |
| Embase | 628 | 11/9/2023 | XYZ |
| APA PsycInfo (EBSCOHost) | 16 | 13/9/2023 | XYZ |
| Cochrane Library | 2763 | 13/9/2023 | XYZ |
| CINAHL Plus with Full Text (EBSCOHost) | 153 | 12/9/2023 | XYZ |
| Web of Science | 2149 | 12/9/2023 | XYZ |
| CNKI | 211 | 12/9/2023 | XYZ |
| Wanfang | 463 | 13/9/2023 | XYZ |
| VIP | 184 | 13/9/2023 | XYZ |

Search Strategy for each source

| PubMed: | |
| --- | --- |
| Search Strategy: | |
| #1 | (((((((((((((((((((((((((wound healing[MeSH Terms]) OR (pressure ulcer[MeSH Terms])) OR (diabetic foot[MeSH Terms])) OR (skin ulcer[MeSH Terms])) OR (leg ulcer[MeSH Terms])) OR (surgical wound[MeSH Terms])) OR (chronic wound*[Title/Abstract])) OR (chronic ulcer*[Title/Abstract])) OR (chronic sore*[Title/Abstract])) OR (nonhealing wound*[Title/Abstract])) OR (chronic ulcer*[Title/Abstract])) OR (nonhealing ulcer*[Title/Abstract])) OR (vascular ulcer*[Title/Abstract])) OR (venous ulcer*[Title/Abstract])) OR (arterial ulcer*[Title/Abstract])) OR (diabetic ulcer*[Title/Abstract])) OR (stasis ulcer*[Title/Abstract])) OR (pressure injur*[Title/Abstract])) OR (pressure sore*[Title/Abstract])) OR (decubitus*[Title/Abstract])) OR (decubitus ulcer*[Title/Abstract])) OR (bed ulcer*[Title/Abstract])) OR (bed sore*[Title/Abstract])) OR (traumatic wound*[Title/Abstract])) OR (stress injur*[Title/Abstract])) OR (malignant wound*[Title/Abstract]) |
| #2 | ((((((((((((((((digital technology[MeSH Terms]) OR (ehealth[Title/Abstract])) OR (ehealth[Title/Abstract])) OR (mobile health[Title/Abstract])) OR (internet[Title/Abstract])) OR (telemedicine[Title/Abstract])) OR (telehealth[Title/Abstract])) OR (remote consultation[Title/Abstract])) OR (software[Title/Abstract])) OR (web-based[Title/Abstract])) OR (website*[Title/Abstract])) OR (phone*[Title/Abstract])) OR (mobile application*[Title/Abstract])) OR (mobile APP*[Title/Abstract])) OR (computer*[Title/Abstract])) OR (text messag*[Title/Abstract])) OR (email*[Title/Abstract]) |
| #3 | ((((((((((randomized controlled trials as topic[MeSH Terms]) OR (random allocation[MeSH Terms])) OR (clinical trials as topic[MeSH Terms])) OR (randomized controlled trial[Publication Type])) OR (controlled clinical trial[Publication Type])) OR (clinical trial[Publication Type])) OR (randomized[Title/Abstract])) OR (placebo[Title/Abstract])) OR (randomly[Title/Abstract])) OR (trial[Title])) OR (groups[Title/Abstract]) |
| #4 | #1 AND #2 AND #3 |

| Embase: | |
| --- | --- |
| Search Strategy: | |
| #1 | 'chronic wound*':ab,ti OR 'chronic ulcer*':ab,ti OR 'chronic sore*':ab,ti OR 'nonhealing wound*':ab,ti OR 'chronic ulcer*':ab,ti OR 'nonhealing ulcer*':ab,ti OR 'vascular ulcer*':ab,ti OR 'venous ulcer*':ab,ti OR 'arterial ulcer*':ab,ti OR 'diabetic ulcer*':ab,ti OR 'stasis ulcer*':ab,ti OR 'pressure injur*':ab,ti OR 'pressure ulcer'/exp OR 'pressure sore*':ab,ti OR 'decubitus*':ab,ti OR 'decubitus ulcer*':ab,ti OR 'bed ulcer*':ab,ti OR 'bed sore*':ab,ti OR 'diabetic foot'/exp OR 'traumatic wound*':ab,ti OR 'stress injur*':ab,ti |
| #2 | 'digital technology':ab,ti OR ehealth:ab,ti OR mhealth:ab,ti OR 'mobile health':ab,ti OR internet:ab,ti OR telemedicine:ab,ti OR telehealth:ab,ti OR 'remote consultation':ab,ti OR software:ab,ti OR 'web based':ab,ti OR website*:ab,ti OR phone*:ab,ti OR 'mobile application*':ab,ti OR 'mobile app*':ab,ti OR computer*:ab,ti OR 'text messag*':ab,ti OR email*:ab,ti |
| #3 | exp randomized controlled trials as topic/ OR exp random allocation/ OR exp clinical trials as topic/ OR randomized.ti,ab. OR placebo.ti,ab. OR randomly.ti,ab. OR trial.ti OR groups.ti,ab. |
| #4 | #1 AND #2 AND #4 |

| APA PsycInfo (EBSCOHost): | |
| --- | --- |
| Search Strategy: | |
| S1 | MH wound healing OR MH pressure ulcer OR MH diabetic foot OR MH skin ulcer OR MH leg ulcer OR MH surgical wound OR TI chronic wound* OR AB chronic wound* OR TI chronic ulcer* OR AB chronic ulcer* OR TI chronic sore* OR AB chronic sore* OR TI nonhealing wound* OR AB nonhealing wound* OR TI chronic ulcer* OR AB chronic ulcer* OR TI nonhealing ulcer* OR AB nonhealing ulcer* OR TI vascular ulcer* OR AB vascular ulcer* OR TI venous ulcer* OR AB venous ulcer* OR TI arterial ulcer* OR AB arterial ulcer* OR TI diabetic ulcer* OR AB diabetic ulcer* OR TI stasis ulcer* OR AB stasis ulcer* OR TI pressure injur* OR AB pressure injur* OR TI pressure sore* OR AB pressure sore* OR TI decubitus* OR AB decubitus* OR TI decubitus ulcer* OR AB decubitus ulcer* OR TI bed ulcer* OR AB bed ulcer* OR TI bed sore* OR AB bed sore* OR TI traumatic wound* OR AB traumatic wound* OR TI malignant wound* OR AB malignant wound* |
| S2 | MH digital technology OR TI ehealth OR AB ehealth OR TI mhealth OR AB mhealth OR TI mobile health OR AB mobile health OR TI internet OR AB internet OR TI telemedicine OR AB telemedicine OR AB telehealth OR TI telehealth OR AB remote consultation OR TI remote consultation OR AB software OR TI software OR AB web-based OR TI web-based OR AB website* OR TI website* OR AB phone OR TI phone OR AB mobile application* OR TI mobile application* OR AB mobile APP* OR TI mobile APP* OR AB computer* OR TI computer* OR AB text messag* OR TI text messag* OR AB email* OR TI email* |
| S3 | MH randomized controlled trial* OR MH clinical trial* OR TI randomized OR TI placebo OR TI randomly OR TI trial OR TI groups OR AB randomized OR AB placebo OR AB randomly OR AB trial OR AB groups |
| S4 | S1 AND S2 AND S3 |

| Cochrane Library (Cochrane Central Register of Controlled Trials): | |
| --- | --- |
| Search Strategy: | |
| 1 | MeSH descriptor: [wound healing] explode all trees OR MeSH descriptor: [pressure ulcer] explode all trees OR MeSH descriptor: [diabetic foot] explode all trees OR MeSH descriptor: [skin ulcer] explode all trees OR MeSH descriptor: [leg ulcer] explode all trees OR MeSH descriptor: [surgical wound] explode all trees OR (chronic wound*): ti,ab,kw OR (chronic ulcer*): ti,ab,kw OR (chronic sore*): ti,ab,kw OR (nonhealing wound*): ti,ab,kw OR (chronic ulcer*): ti,ab,kw OR (chronic sore*): ti,ab,kw OR (nonhealing wound*): ti,ab,kw OR (chronic ulcer*): ti,ab,kw OR (nonhealing ulcer*): ti,ab,kw OR (vascular ulcer*): ti,ab,kw OR (venous ulcer*): ti,ab,kw OR (arterial ulcer*): ti,ab,kw OR (diabetic ulcer*): ti,ab,kw OR (diabetic ulcer*): ti,ab,kw OR (stasis ulcer*): ti,ab,kw OR (pressure injur*): ti,ab,kw OR (pressure sore*): ti,ab,kw OR (decubitus*): ti,ab,kw OR (decubitus ulcer*): ti,ab,kw OR (bed ulcer*): ti,ab,kw OR (bed sore*): ti,ab,kw OR (traumatic wound*): ti,ab,kw OR (stress injur*): ti,ab,kw OR ( malignant wound*): ti,ab,kw |
| 2 | MeSH descriptor: [[digital technology](https://www.ncbi.nlm.nih.gov/mesh/2051971)] explode all trees OR (ehealth): ti,ab,kw OR (mhealth): ti,ab,kw OR (mobile health): ti,ab,kw OR (internet): ti,ab,kw OR (telemedicine): ti,ab,kw OR (telehealth): ti,ab,kw OR (remote consultation): ti,ab,kw OR (software): ti,ab,kw OR (web-based): ti,ab,kw OR (website*): ti,ab,kw OR (phone*): ti,ab,kw OR (mobile application*): ti,ab,kw OR (mobile APP*): ti,ab,kw OR (computer*): ti,ab,kw OR (text messag*): ti,ab,kw OR (email*): ti,ab,kw |
| 3 | (digital behavio*): ti,ab,kw OR (digital intervention*): ti,ab,kw OR (ehealth): ti,ab,kw OR (mhealth): ti,ab,kw OR (mobile health): ti,ab,kw OR (internet): ti,ab,kw OR (telemedicine): ti,ab,kw OR (telehealth): ti,ab,kw OR (software): ti,ab,kw OR (web-based): ti,ab,kw OR (website*): ti,ab,kw OR (phone*): ti,ab,kw OR (mobile application*): ti,ab,kw OR (mobile APP*): ti,ab,kw OR (computer*): ti,ab,kw OR (text messag*): ti,ab,kw OR (email*): ti,ab,kw OR (wearable electronic device*): ti,ab,kw OR (fitness tracker*): ti,ab,kw OR (fitbit*): ti,ab,kw OR (activity tracker*): ti,ab,kw OR (video game*): ti,ab,kw OR (virtual realit*): ti,ab,kw OR (augmented realit*): ti,ab,kw OR (exergam*): ti,ab,kw |
| 4 | 1 AND 2 AND 3 |

| CINAHL Plus with Full Text (EBSCOHost): | |
| --- | --- |
| Search Strategy: | |
| S1 | MH wound healing OR MH pressure ulcer OR MH diabetic foot OR MH skin ulcer OR MH leg ulcer OR MH surgical wound OR TI chronic wound* OR AB chronic wound* OR TI chronic ulcer* OR AB chronic ulcer* OR TI chronic sore* OR AB chronic sore* OR TI nonhealing wound* OR AB nonhealing wound* OR TI chronic ulcer* OR AB chronic ulcer* OR TI nonhealing ulcer* OR AB nonhealing ulcer* OR TI vascular ulcer* OR AB vascular ulcer* OR TI venous ulcer* OR AB venous ulcer* OR TI arterial ulcer* OR AB arterial ulcer* OR TI diabetic ulcer* OR AB diabetic ulcer* OR TI stasis ulcer* OR AB stasis ulcer* OR TI pressure injur* OR AB pressure injur* OR TI pressure sore* OR AB pressure sore* OR TI decubitus* OR AB decubitus* OR TI decubitus ulcer* OR AB decubitus ulcer* OR TI bed ulcer* OR AB bed ulcer* OR TI bed sore* OR AB bed sore* OR TI traumatic wound* OR AB traumatic wound* OR TI malignant wound* OR AB malignant wound* |
| S2 | MH digital technology OR TI ehealth OR AB ehealth OR TI mhealth OR AB mhealth OR TI mobile health OR AB mobile health OR TI internet OR AB internet OR TI telemedicine OR AB telemedicine OR AB telehealth OR TI telehealth OR AB remote consultation OR TI remote consultation OR AB software OR TI software OR AB web-based OR TI web-based OR AB website* OR TI website* OR AB phone OR TI phone OR AB mobile application* OR TI mobile application* OR AB mobile APP* OR TI mobile APP* OR AB computer* OR TI computer* OR AB text messag* OR TI text messag* OR AB email* OR TI email* |
| S3 | MH randomized controlled trial* OR MH clinical trial* OR TI randomized OR TI placebo OR TI randomly OR TI trial OR TI groups OR AB randomized OR AB placebo OR AB randomly OR AB trial OR AB groups |
| S4 | S1 AND S2 AND S3 |

| Web of science: | |
| --- | --- |
| Search Strategy: | |
| 1 | TS=(wound healing) OR TS=(pressure ulcer) OR TS=(diabetic foot) OR TS=(skin ulcer) OR TS=(leg ulcer) OR TS=(surgical wound) OR TI=(chronic wound*) OR TI=(chronic ulcer*) OR TI=(chronic sore*) OR TI=(nonhealing wound*) OR TI=(chronic ulcer*) OR TI=(nonhealing ulcer*) OR TI=(vascular ulcer*) OR TI=(venous ulcer*) OR TI=(arterial ulcer*) OR TI=(diabetic ulcer*) OR TI=(stasis ulcer*) OR TI=(pressure injur*) OR TI=(pressure sore*) OR TI=(decubitus*) OR TI=(decubitus ulcer*) OR TI=(bed ulcer*) OR TI=(bed sore*) OR TI=(traumatic wound*) OR TI=(stress injur*) OR TI=(malignant wound*) OR AB=(chronic wound*) OR AB=(chronic ulcer*) OR AB=(chronic sore*) OR AB=(nonhealing wound*) OR AB=(chronic ulcer*) OR AB=(nonhealing ulcer*) OR AB=(vascular ulcer*) OR AB=(venous ulcer*) OR AB=(arterial ulcer*) OR AB=(diabetic ulcer*) OR AB=(stasis ulcer*) OR AB=(pressure injur*) OR AB=(pressure sore*) OR AB=(decubitus*) OR AB=(decubitus ulcer*) OR AB=(bed ulcer*) OR AB=(bed sore*) OR AB=(traumatic wound*) OR AB=(stress injur*) OR AB=(malignant wound*) |
| 2 | TS=([digital technology](https://www.ncbi.nlm.nih.gov/mesh/2051971)) OR TI=(ehealth) OR TI=(mhealth) OR TI=(mobile health) OR TI=(internet) OR TI=(telemedicine) OR TI=(telehealth) OR TI=(remote consultation) OR TI=(software) OR TI=(web-based) OR TI=(website*) OR TI=(phone*) OR TI=(mobile application*) OR TI=(mobile app*) OR TI=(computer*) OR TI=(text messag*) OR TI=(email*) OR AB=(ehealth) OR AB=(mhealth) OR AB=(mobile health) OR AB=(internet) OR AB=(telemedicine) OR AB=(telehealth) OR AB=(remote consultation) OR AB=(software) OR AB=(web-based) OR AB=(website*) OR AB=(phone*) OR AB=(mobile application*) OR AB=(mobile app*) OR AB=(computer*) OR AB=(text messag*) OR AB=(email*) |
| 3 | TI=(randomized controlled trial*) OR TI=(controlled trial*) OR TI=(randomized) OR TI=(placebo) OR TI=(randomly) OR TI=(trial) OR TI=(groups) OR AB=(randomized controlled trial*) OR AB=(controlled trial*) OR AB=(randomized) OR AB=(placebo) OR AB=(randomly) OR AB=(trial) OR AB=(groups) |
| 4 | 1 AND 2 AND 3 |

| CNKI: | |
| --- | --- |
| Search Strategy: | |
| 1 | SU=慢性伤口 OR TKA=慢性创伤 OR TKA=慢性溃疡 OR TKA=静脉性溃疡 OR TKA=动脉性溃疡OR TKA=糖尿病性溃疡 OR TKA=糖尿病足 OR TKA=创伤性伤口 OR TKA=压力性损伤 OR TKA=压疮 OR TKA=褥疮 |
| 2 | SU=数字健康 OR SU=数字医疗 OR TKA=电子健康 OR TKA=移动健康 OR TKA=互联网 OR TKA=远程医疗 OR TKA=远程康复 OR TKA=远程咨询 OR TKA=应用程序 OR TKA=网站 OR TKA=电话 OR TKA=电脑 OR TKA=短信 OR TKA=邮件 |
| 3 | SU=随机对照试验 OR SU=临床对照试验 OR TKA=随机 OR TKA=对照 OR TKA=试验 OR TKA=分组 |
| 4 | 1 AND 2 AND 3 AND 4 |

| Wanfang: | |
| --- | --- |
| Search Strategy: | |
| 1 | 主题:(慢性伤口) OR 摘要:(慢性创伤 OR 慢性溃疡 OR 静脉性溃疡 OR 动脉性溃疡 OR 糖尿病性溃疡 OR 糖尿病足 OR 创伤性伤口 OR 压力性损伤 OR 褥疮 OR 压疮) |
| 2 | 主题:(数字健康 OR 数字医疗) OR 摘要:(电子健康 OR 移动健康 OR 互联网 OR远程医疗 OR 远程康复 OR 远程咨询 OR 应用程序 OR 网站 OR 电话 OR 电脑 OR 短信 OR 邮件） |
| 3 | 主题:(随机对照试验 OR 临床对照试验）OR 摘要:(随机 OR对照 OR 试验 OR分组） |
| 4 | 1 AND 2 AND 3 AND 4 |

| VIP: | |
| --- | --- |
| Search Strategy: | |
| 1 | M=慢性伤口 OR R=(慢性创伤 OR 慢性溃疡 OR 静脉性溃疡 OR 动脉性溃疡 OR 糖尿病性溃疡 OR 糖尿病足 OR 创伤性伤口 OR 压力性损伤 OR 压疮 OR 褥疮) |
| 2 | M=(数字健康 OR 数字医疗) OR R=(电子健康 OR 移动健康 OR 互联网 OR远程医疗 OR 远程康复 OR 远程咨询 OR 应用程序 OR 网站 OR 电话 OR 电脑 OR 短信 OR 邮件) |
| 3 | M=(随机对照试验 OR临床对照试验) OR R=(随机 OR 对照 OR 试验 OR 分组) |
| 4 | 1 AND 2 AND 3 AND 4 |
