# Supplementary material for: Effectiveness of Telemedicine on Wound-Related and Patient-Reported Outcomes in Patients With Chronic Wounds: Systematic Review and Meta-Analysis
Source: JMIR Mhealth Uhealth. 2025 Jun 10;13:e58553. doi: 10.2196/58553 (PMC12173094; doi:10.2196/58553)
Supplement: Multimedia Appendix 2 [file mhealth-v13-e58553-s002.doc]

| **Author, year** | **Country** | **Chronic wound type** | **Mean age**  **(years)**  **TMsG/CG** | **TMs group** | | **Control group** | | **Intervention**  **duration**  **(months)** | **Outcomes** |
| --- | --- | --- | --- | --- | --- | --- | --- | --- | --- |
|  |  |  |  | **Description** | **N** | **Description** | **N** |  |  |
| Arora et al (2017) [43] | Australia | Pressure injury | 35.0/35.0 | - Telephone: weekly advice by telephone for 12 weeks about the management of pressure ulcers from a trained healthcare professional | 60 | Usual care | 60 | 3 | ①, ④, ⑥, ⑦, ⑨ |
| Beibei (2019) [41] | China | Mixed | 58.63/59.4 | - WeChat and cloud consulting room: the TMs group used WeChat and cloud consulting room as "Internet +" carriers. The intervention included health education, wound dressing reminder, and wound disease response | 32 | Usual care | 30 | 2 | ①, ⑥, ⑧ |
| Dobke et al (2008) [52] | USA | Mixed | 54.9/53.9 | - The telemedicine consult included: wound assessment, rationale for the suggested wound management with emphasis on wound risk projections, and prevention and benefits of surgical intervention | 15 | Face-to-face consult | 15 | 0.5 | ⑨ |
| Haiyan et al (2021) [32] | China | Pressure injury | 77.5/75.5 | - Remote robot and WeChat: Remote robot work meetings once a month; Remote wound care clinics were set up in the community, and robots were connected to the community for intervention. Home remote guidance, using WeChat video call remote consultation; Remote health education classroom; The wound nurse visited the hospital once a month | 60 | Usual care | 60 | 6 | ①, ⑥ |
| Hua et al (2022) [28] | China | Mixed | 68.2/66.6 | - Wechat group: Establish a wound group, send thematic content to the WeChat group, answer questions in the medical group, share experiences inpatient group, and conduct family follow-up visits twice a month by nursing staff; One to one, one to many collaborative nursing, patients WeChat send wound photos, nursing staff analysis and guidance, reasonable arrangement of dressing change frequency | 48 | Usual care | 48 | 2 | ①, ⑤, ⑥ |
| Huanli et al (2014) [46] | China | Pressure injury | 63.7 | - QQ group and Weibo: create QQ groups, Weibo, timely update health knowledge, patient communication, if necessary, door-to-door guidance | 32 | Usual care | 31 | 2 | ① |
| Iversen et al (2020) [29] | Norway | Diabetic foot ulcer | 66.3/64.7 | - Computer networking platform and mobile phone: an interactive web-based ulcer record and a mobile phone enabling counseling and communication between the community nurses and specialist healthcare | 78 | Standard care | 78 | 12 | ①, ②, ③, ⑥, ⑦, ⑧ |
| Jin (2021) [31] | China | Mixed | 67.9/67.8 | - Wechat group: remote chronic wound care, one-to-one or one-to-many collaborative care | 54 | Usual care | 54 | 2 | ①, ⑤ |
| Jing et al (2022) [26] | China | Diabetic foot ulcer | 59.2/56.7 | - Wechat public platform, communication group: 5A nursing model based on Internet platform including asking, advising, assessing, assisting, and arrange | 41 | Usual care | 41 | 12 | ⑥, ⑨ |
| Jingwen et al (2022) [27] | China | Diabetic foot ulcer | 52.1/52.4 | - Diabetes foot care application and WeChat group: Formed a professional team, developed an APP, established a WeChat group, established a diabetic foot care clinic, established a medical cooperation group, and carried out home care services | 48 | Usual care | 48 | 6 | ⑥ |
| Rasmussen et al (2015) [45] | Denmark | Diabetic foot ulcer | 66.8/66.7 | - Telephone or online written consultation: The per-protocol telemedical monitoring consisted of two consultations in the patient's own home and one consultation at the outpatient clinic | 206 | Standard care | 195 | 12 | ①, ②, ③, ④ |
| Santamaria et al (2004) [53] | Australia | Mixed | 63.5/49.5 | - The Alfred/Medseed Wound Imaging System: TMs group patients had their digital records transmitted to Perth every 2 weeks for remote review by a wound care consultant; these were then returned to their treating clinician with wound management advice | 50 | Standard care | 43 | 12 | ①, ②, ③, ④ |
| Smith-Strøm et al (2018) [42] | Norway | Diabetic foot ulcer | 70.0/65.0 | - The TM application: The TM application consisted of an interactive Web-based ulcer record and a mobile phone, enabling counseling and communication between the community nurses and specialist healthcare | 94 | Standard care | 88 | 12 | ①, ②, ③,⑨ |
| Stern et al (2014) [48] | Canada | Pressure injury | 83.0/81.0 | - Email, telephone, or video link: An advanced practice nurse with expertise in skin and wound care visited intervention facilities to educate staff on pressure ulcer prevention and treatment, supported by an off-site hospital-based expert multi-disciplinary wound care team via email, telephone, or video link as needed | 101 | Usual care | 80 | 12 | ①, ④, ⑤ |
| Téot et al (2020) [30] | France | Mixed | 72.0/70.3 | - A dedicated cloud-based software program: The publicly funded network of nurses and physicians highly experienced in wound healing was used to provide wound care recommendations via telemedicine for the study | 110 | Usual care | 41 | 6 | ①, ③, ④ |
| Terry et al (2009) [51] | USA | Mixed | 58.4/57.5 | - Each study nurse in the TM group was provided with a Nikon Coolpix 4500 digital camera, a Fujitsu B series laptop computer, and a tripod: weekly visits with TM and wound care specialist consults | 40 | Usual care | 35 | 16 | ①, ④ |
| Ting et al (2014) [50] | China | Mixed | 47.4 | - MMS platform: construct patient files, upload wound MMS photos every week, provide remote feedback from experts, and conduct wound treatment according to opinions | 25 | Usual care | 12 | 6 | ①, ⑤ |
| Vowden et al (2013) [49] | UK | Mixed | 79.5/82.7 | - Remote support： wound images were taken using a smartphone camera and these data were electronically linked to the digital paper form. Once all the data were collected, the wound images and information were uploaded together to a secure server for assessment by the remote nurse consultant. | 17 | Standard care | 9 | 6 | ①, ③ |
| Weiwei et al (2014) [47] | China | Venous ulcer | 59.7/60.8 | - Phone, WeChat or QQ group, mailbox: call every 2 weeks to inquire about the wound; Use mobile phones, WeChat, or QQ to upload photos, according to the wound assessment and tracking; Outpatient visits at the end of each month; Answer questions online every Monday, Wednesday, and Friday | 30 | Usual care | 30 | 3 | ①, ⑨ |
| Xiaoping et al (2016) [44] | China | Pressure injury | 77.2/79.4 | - Phone or Internet platform: "Hospital-community" combined pressure ulcer care teams provide continuity of care | 50 | Usual care | 50 | 6 | ① |
| Xuemei et al (2023) [25] | China | Mixed | 58.7/58.1 | - WeChat mini program: Establish an integrated service team, establish an online nurse database, online nurse specialist training, chronic wound care service (household service), and security protection | 53 | Usual care | 53 | 2 | ①, ⑨ |
| Yumei et al (2020) [40] | China | Mixed | 71.5/74.3 | - Internet nursing platform: The hospital's Internet nursing platform makes appointments and consultations, and the network nurses provide regular on-site services | 26 | Usual care | 26 | 1 | ①, ⑥, ⑨ |

①: wound healing; ②: amputation rate; ③: mortality; ④: economic evaluation; ⑤: pain; ⑥: quality of life; ⑦: depression; ⑧: anxiety; ⑨: patient’s satisfaction
