# Supplementary figures and images for: Effectiveness of Telemedicine on Wound-Related and Patient-Reported Outcomes in Patients With Chronic Wounds: Systematic Review and Meta-Analysis
Source: JMIR Mhealth Uhealth. 2025 Jun 10;13:e58553. doi: 10.2196/58553 (PMC12173094; doi:10.2196/58553)

**
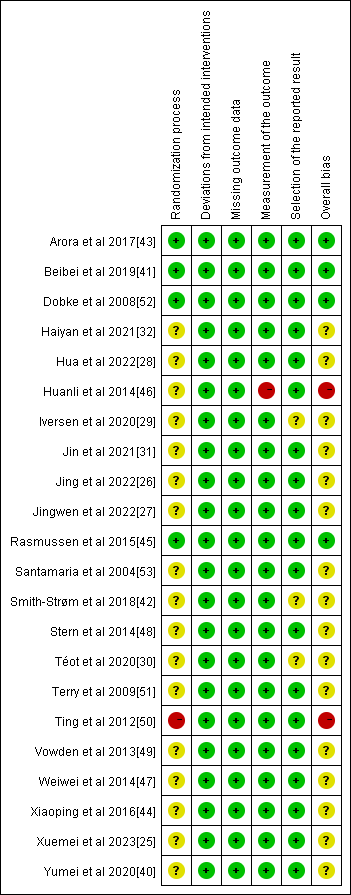
**

Supplement: Multimedia Appendix 3 [file mhealth-v13-e58553-s003.doc]

**
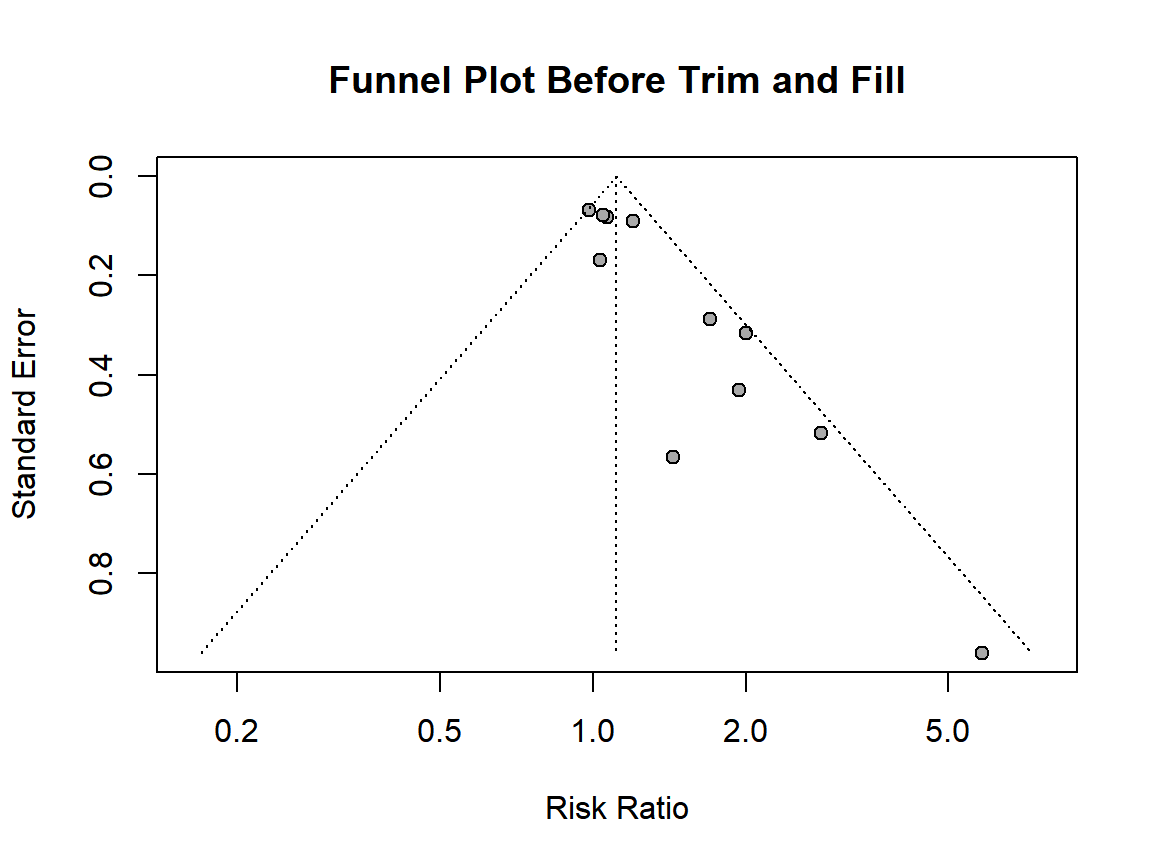
**

Supplement: Multimedia Appendix 6 [file mhealth-v13-e58553-s006.doc]

**
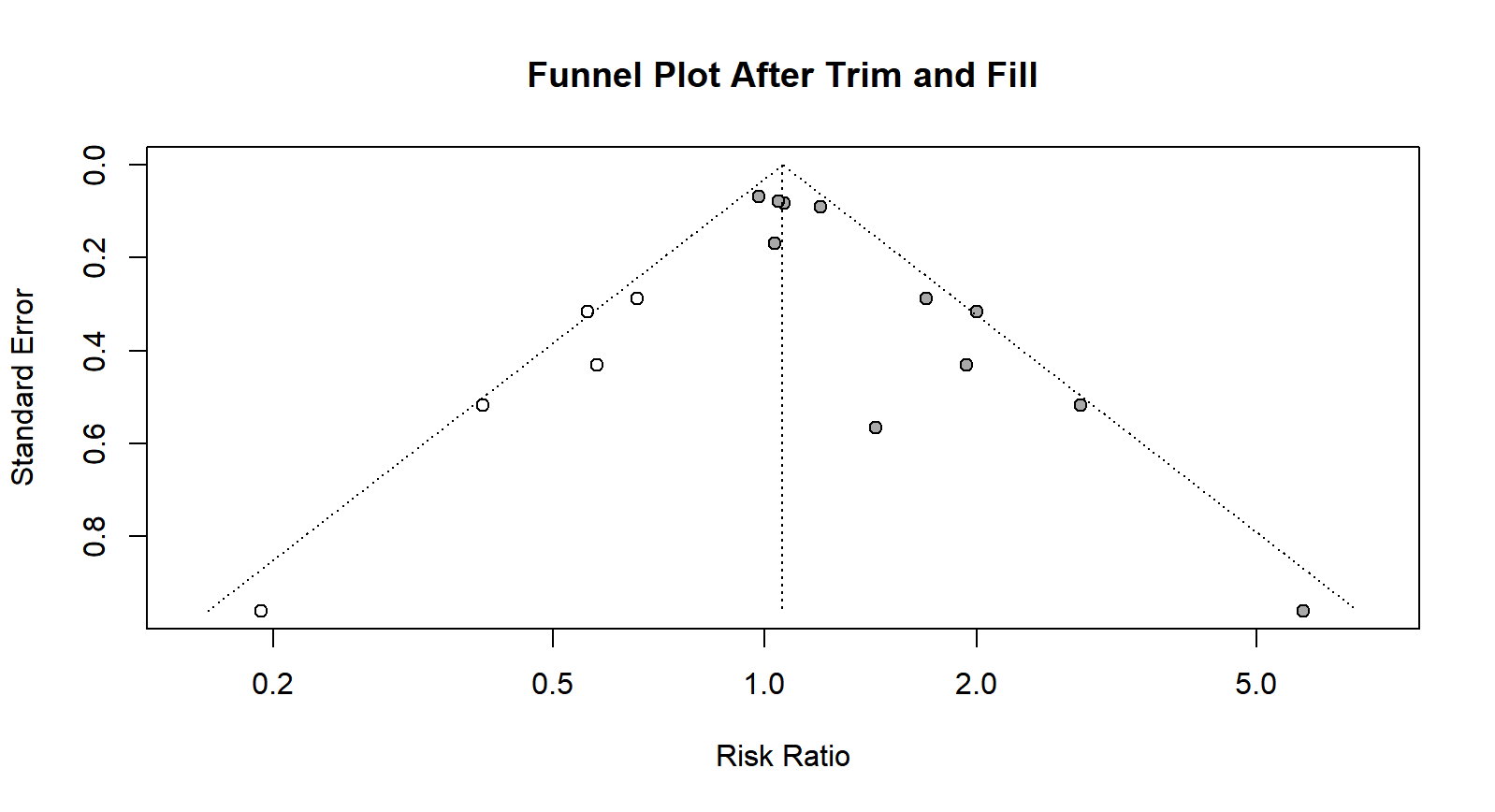
**

Supplement: Multimedia Appendix 7 [file mhealth-v13-e58553-s007.doc]
