# Supplementary material for: Effectiveness of Telemedicine on Wound-Related and Patient-Reported Outcomes in Patients With Chronic Wounds: Systematic Review and Meta-Analysis
Source: JMIR Mhealth Uhealth. 2025 Jun 10;13:e58553. doi: 10.2196/58553 (PMC12173094; doi:10.2196/58553)
Supplement: Multimedia Appendix 4 [file mhealth-v13-e58553-s004.doc]

| **Certainty assessment** | | | | | | **No. of participants** | | **Effect**  **(95% CI)** | **Certainty** |
| --- | --- | --- | --- | --- | --- | --- | --- | --- | --- |
| **Participants (studies)** | **Limitations** | **Inconsistency** | **Indirectness** | **Imprecision** | **Other considerations** | **Telemedicine** | **Control** |
| **Healing rate** | | | | | | | | | |
| 1425  (11 RCTs) | not serious | not serious | not serious | serious ^a^ | serious ^b^ | 778 | 647 | RR (95% CI): 1.16  (1.02 to 1.33) | ⨁⨁◯◯ Low |
| **Healing score** | | | | | | | | | |
| 668  (7 RCTs) | not serious | serious ^c^ | not serious | not serious | none | 335 | 333 | SMD (95% CI): −1.46  (−2.27 to −0.66) | ⨁⨁⨁◯ Moderate |
| **Healing time** | | | | | | | | | |
| 577  (6 RCTs) | not serious | serious ^c^ | not serious | serious ^a^ | none | 290 | 287 | SMD (95% CI): −0.47  (−0.92 to −0.02 ) | ⨁⨁◯◯ Low |
| **Amputation rate** | | | | | | | | | |
| 832 (4 RCTs) | not serious | not serious | not serious | serious ^a^ | none | 428 | 404 | RR (95% CI): 0.52 (0.31 to 0.88) | ⨁⨁⨁◯ Moderate |
| **Mortality** | | | | | | | | | |
| 1009  (6 RCTs) | not serious | not serious | not serious | serious ^a^ | none | 555 | 454 | RR (95% CI): 0.94  (0.41 to 2.11) | ⨁⨁⨁◯ Moderate |
| **Pain** | | | | | | | | | |
| 204  (2 RCTs) | not serious | not serious | not serious | serious ^d^ | none | 102 | 102 | SMD (95% CI): −0.62  (−0.19 to −0.34) | ⨁⨁⨁◯ Moderate |
| **Quality of life** | | | | | | | | | |
| 492  (4 RCTs) | not serious | serious ^c^ | not serious | not serious | none | 246 | 246 | SMD (95% CI): 1.90  (0.32 to 3.48 ) | ⨁⨁⨁◯ Moderate |
| **Depression** | | | | | | | | | |
| 276  (2 RCTs) | not serious | not serious | not serious | serious ^d^ | none | 138 | 138 | SMD (95% CI): −0.03  (−0.27 to 0.20) | ⨁⨁⨁◯ Moderate |
| **Anxiety** | | | | | | | | | |
| 218  (2 RCTs) | not serious | serious ^c^ | not serious | serious ^d^ | none | 110 | 108 | SMD (95% CI): −1.25  (−3.62 to 1.13) | ⨁⨁◯◯ Low |
| **Patient’s satisfaction rate** | | | | | | | | | |
| 218  (3 RCTs) | not serious | serious ^c^ | not serious | serious ^d^ | none | 109 | 109 | RR (95% CI): 1.26  (0.92 to 1.73) | ⨁⨁◯◯ Low |
| **Patient’s satisfaction score** | | | | | | | | | |
| 384  (3 RCTs) | not serious | serious ^c^ | not serious | serious ^d^ | none | 195 | 189 | SMD (95% CI): 0.85  (−0.08 to 1.79) | ⨁⨁◯◯ Low |

**CI:** Confidence interval**; SMD:** Standardized mean differences**; RR:** Risk ratio

#### Explanations

1. Downgraded one level for imprecision due to wide confidence intervals.
2. Downgraded one level for other considerations due to publication bias.
3. Downgraded one level for inconsistency due to wide variance of point estimates across studies (high heterogeneity).
4. Downgraded one level for imprecision due to small sample sizes.
