# Supplementary material for: Effectiveness of Telemedicine on Wound-Related and Patient-Reported Outcomes in Patients With Chronic Wounds: Systematic Review and Meta-Analysis
Source: JMIR Mhealth Uhealth. 2025 Jun 10;13:e58553. doi: 10.2196/58553 (PMC12173094; doi:10.2196/58553)
Supplement: Multimedia Appendix 5 [file mhealth-v13-e58553-s005.doc]

| **Outcomes** | **No. of**  **studies** | **No. of**  **participants** | **Meta-analysis** | | | | **Heterogeneity** | |
| --- | --- | --- | --- | --- | --- | --- | --- | --- |
|  |  |  | **RR/SMD** | **95%CI** | | ***P* value** | ***I*^2^** | ***PQ*** |
| **Healing rate** |  |  |  |  |  |  |  |  |
| Study duration |  |  |  |  |  |  |  |  |
| ≤ 6 months | 7 | 505 | 1.47 | 1.07 | 2.00 | .02^a^ | 51% | .06 |
| 6–12 months | 3 | 739 | 1.03 | 0.94 | 1.13 | .56 | 0% | .70 |
| > 12 months | 1 | 181 | 1.70 | 0.97 | 2.98 | .07 | n/a | n/a |
| Number of telemedicine types |  |  |  |  |  |  |  |  |
| 1 | 4 | 320 | 1.18 | 0.94 | 1.47 | .15 | 20% | .29 |
| ≥ 2 | 7 | 1105 | 1.17 | 0.99 | 1.40 | .07 | 61% | .02 |
| Average age |  |  |  |  |  |  |  |  |
| 18-60 | 4 | 265 | 1.61 | 0.98 | 2.66 | .06 | 59% | .06 |
| ≥ 60 | 7 | 1160 | 1.08 | 0.95 | 1.22 | .25 | 39% | .13 |
| Type of chronic wounds |  |  |  |  |  |  |  |  |
| Diabetic foot ulcer | 3 | 739 | 1.03 | 0.94 | 1.12 | .56 | 0% | .70 |
| Pressure injury | 2 | 244 | 1.77 | 1.11 | 2.82 | .02^a^ | 0% | .80 |
| Venous ulcer | 1 | 60 | 2.00 | 1.08 | 3.72 | .03^a^ | n/a | n/a |
| Mixed | 5 | 382 | 1.30 | 0.94 | 1.79 | .11 | 46% | .12 |
| Telemedicine in conjunction with a face-to-face component |  |  |  |  |  |  |  |  |
| Yes | 7 | 1055 | 1.22 | 1.02 | 1.47 | .03^a^ | 63% | .01 |
| No | 4 | 370 | 1.10 | 0.87 | 1.38 | .43 | 24% | .26 |
| Telemedicine including communication components |  |  |  |  |  |  |  |  |
| Yes | 9 | 1362 | 1.14 | 1.00 | 1.29 | .04^a^ | 50% | .04 |
| No | 2 | 63 | 2.38 | 0.60 | 9.47 | .22 | 42% | .19 |
| **Healing score** |  |  |  |  |  |  |  |  |
| Number of telemedicine types |  |  |  |  |  |  |  |  |
| 1 | 4 | 386 | −0.59 | −0.80 | −0.39 | < .001^a^ | 0% | .91 |
| ≥ 2 | 3 | 282 | −2.79 | −5.21 | −0.36 | .02^a^ | 98% | < .001 |
| Average age |  |  |  |  |  |  |  |  |
| 18-60 | 3 | 288 | −0.75 | −1.15 | −0.35 | < .001^a^ | 61% | .07 |
| ≥ 60 | 4 | 380 | −2.07 | −3.66 | −0.49 | .01^a^ | 98% | < .001 |
| Type of chronic wounds |  |  |  |  |  |  |  |  |
| Pressure injury | 3 | 340 | −2.53 | −4.68 | −0.38 | .02^a^ | 98% | < .001 |
| Mixed | 4 | 328 | −0.75 | −1.05 | −0.45 | < .001^a^ | 42% | .16 |
| Telemedicine in conjunction with a face-to-face component |  |  |  |  |  |  |  |  |
| Yes | 5 | 440 | −1.89 | −3.14 | −0.65 | .003^a^ | 97% | < .001 |
| No | 2 | 228 | −0.56 | −0.83 | −0.30 | < .001^a^ | 0% | .98 |
| **Healing time** |  |  |  |  |  |  |  |  |
| Number of telemedicine types |  |  |  |  |  |  |  |  |
| 1 | 4 | 335 | −0.42 | −0.97 | 0.12 | .13 | 83% | < .001 |
| ≥ 2 | 2 | 242 | −0.60 | −1.82 | 0.62 | .34 | 93% | < .001 |
| Average age |  |  |  |  |  |  |  |  |
| 18-60 | 1 | 60 | −1.24 | −1.80 | −0.69 | < .001^a^ | n/a | n/a |
| ≥ 60 | 5 | 517 | −0.33 | −0.77 | 0.11 | < .001^a^ | 83% | .001 |
| Type of chronic wounds |  |  |  |  |  |  |  |  |
| Diabetic foot ulcer | 1 | 182 | 0.00 | −0.29 | 0.29 | 1.00 | n/a | n/a |
| Venous ulcer | 1 | 60 | −1.24 | −1.80 | −0.69 | < .001^a^ | n/a | n/a |
| Mixed | 4 | 335 | −0.42 | −0.97 | 0.12 | .13 | 83% | < .001 |
| Telemedicine in conjunction with a face-to-face component |  |  |  |  |  |  |  |  |
| Yes | 4 | 390 | −0.66 | −1.25 | −0.08 | .03^a^ | 86% | .03 |
| No | 2 | 187 | −0.10 | −1.02 | 0.82 | .83 | 90% | .83 |
| **Amputation rate** |  |  |  |  |  |  |  |  |
| Number of telemedicine types |  |  |  |  |  |  |  |  |
| 1 | 1 | 93 | 0.14 | 0.02 | 1.14 | .07 | n/a | n/a |
| ≥ 2 | 3 | 739 | 0.60 | 0.39 | 0.93 | .02^a^ | 3% | .36 |
| Type of chronic wounds |  |  |  |  |  |  |  |  |
| Diabetic foot ulcer | 3 | 739 | 0.60 | 0.39 | 0.93 | .02^a^ | 3% | .36 |
| Mixed | 1 | 93 | 0.14 | 0.02 | 1.14 | .07 | n/a | n/a |
| Telemedicine including communication components |  |  |  |  |  |  |  |  |
| Yes | 3 | 739 | 0.60 | 0.39 | 0.93 | .02^a^ | 3% | .36 |
| No | 1 | 93 | 0.14 | 0.02 | 1.14 | .07 | n/a | n/a |
| **Mortality** |  |  |  |  |  |  |  |  |
| Study duration |  |  |  |  |  |  |  |  |
| ≤ 6 months | 2 | 177 | 0.64 | 0.16 | 2.55 | .53 | 15% | .28 |
| > 12 months | 4 | 832 | 1.11 | 0.36 | 3.38 | .86 | 43% | .16 |
| Number of telemedicine types |  |  |  |  |  |  |  |  |
| 1 | 3 | 270 | 0.51 | 0.16 | 1.64 | .26 | 0% | .41 |
| ≥ 2 | 3 | 739 | 1.39 | 0.43 | 4.48 | .58 | 48% | .15 |
| Type of chronic wounds |  |  |  |  |  |  |  |  |
| Diabetic foot ulcer | 2 | 583 | 2.26 | 0.28 | 18.41 | .45 | 68% | .08 |
| Mixed | 4 | 426 | 0.63 | 0.27 | 1.48 | .29 | 0% | .57 |
| Telemedicine in conjunction with a face-to-face component |  |  |  |  |  |  |  |  |
| Yes | 2 | 583 | 2.26 | 0.28 | 18.41 | .45 | 68% | .08 |
| No | 4 | 426 | 0.63 | 0.27 | 1.48 | .29 | 0% | .57 |
| Telemedicine including communication components |  |  |  |  |  |  |  |  |
| Yes | 4 | 890 | 1.02 | 0.37 | 2.81 | .97 | 47% | .13 |
| No | 2 | 119 | 0.68 | 0.08 | 5.81 | .72 | 31% | .23 |
| **Quality of life** |  |  |  |  |  |  |  |  |
| Average age |  |  |  |  |  |  |  |  |
| 18-60 | 2 | 216 | 3.15 | −1.93 | 8.23 | .22 | 99% | < .001 |
| ≥ 60 | 2 | 276 | 0.76 | −1.02 | 2.54 | .40 | 98% | < .001 |
| Study duration |  |  |  |  |  |  |  |  |
| ≤ 6 months | 3 | 336 | 2.62 | 0.51 | 4.72 | .01^a^ | 98% | < .001 |
| > 12 months | 1 | 156 | −0.14 | −0.46 | 0.17 | .37 | n/a | n/a |
| Number of telemedicine types |  |  |  |  |  |  |  |  |
| 1 | 1 | 120 | 0.57 | 0.21 | 0.94 | .002^a^ | n/a | n/a |
| ≥ 2 | 3 | 372 | 2.39 | −0.07 | 4.84 | .06 | 99% | < .001 |
| Type of chronic wounds |  |  |  |  |  |  |  |  |
| Diabetic foot ulcer | 2 | 252 | 2.79 | −2.99 | 8.57 | .34 | 99% | < .001 |
| Pressure injury | 2 | 240 | 1.12 | 0.04 | 2.20 | .04^a^ | 93% | < .001 |
| Telemedicine in conjunction with a face-to-face component |  |  |  |  |  |  |  |  |
| Yes | 2 | 252 | 2.79 | −2.99 | 8.57 | .34 | 99% | < .001 |
| No | 2 | 240 | 1.12 | 0.04 | 2.20 | .04^a^ | 93% | < .001 |
| **SF-36** |  |  |  |  |  |  |  |  |
| Average age |  |  |  |  |  |  |  |  |
| 18-60 | 2 | 144 | 1.35 | 0.80 | 1.89 | < .001^a^ | 55% | .14 |
| ≥ 60 | 2 | 148 | 0.72 | 0.39 | 1.05 | < .001^a^ | 0% | .92 |
| Study duration |  |  |  |  |  |  |  |  |
| ≤ 6 months | 3 | 210 | 0.81 | 0.53 | 1.10 | < .001^a^ | 0% | .56 |
| > 12 months | 1 | 82 | 1.62 | 1.12 | 2.12 | < .001^a^ | n/a | n/a |
| Number of telemedicine types |  |  |  |  |  |  |  |  |
| 1 | 2 | 148 | 0.72 | 0.39 | 1.05 | < .001^a^ | 0% | .92 |
| ≥ 2 | 2 | 144 | 1.35 | 0.80 | 1.89 | < .001^a^ | 55% | .14 |
| Type of chronic wounds |  |  |  |  |  |  |  |  |
| Diabetic foot ulcer | 1 | 82 | 1.62 | 1.12 | 2.12 | < .001^a^ | n/a | n/a |
| Mixed | 3 | 210 | 0.81 | 0.53 | 1.10 | < .001^a^ | 0% | .56 |

a: *P* < .05
